# Personality Characteristics and Oral Health-Related Quality of Life in an Iranian Adult Population

**DOI:** 10.1155/2021/6619123

**Published:** 2021-01-31

**Authors:** Naimeh Hasheminejad, Hamidreza Hajizamani, Mohammadi Tayebeh Malek, William Murray Thomson

**Affiliations:** ^1^Oral and Dental Disease Research Center, Kerman Social Determinants on Oral Health Research Center, Kerman University of Medical Sciences, Kerman, Iran; ^2^Research Center for Science and Technology in Medicine, Department of Dental Biomaterials, School of Dentistry, Tehran University of Medical Sciences, Tehran, Iran; ^3^Social Determinants of Health Research Center, Institute for Futures Studies in Health, Department of DentalPublic Health, Kerman University of Medical Sciences, Kerman, Iran; ^4^Sir John Walsh Research Institute, The University of Otago, Dunedin, New Zealand

## Abstract

Self-rated oral health and oral health-related quality of life is known to be influenced by various personality characteristics. The aim of this study was to understand how personality characteristics affect oral health-related quality of life ratings in an Iranian adult population. The study included 443 adult participants recruited from a public dental clinic in Kerman, southeast of Iran. The Oral Health Impact Profile-14 questionnaire was used to assess oral health-related quality of life. Personality traits were determined using the 20-item Positive Affectivity Negative Affectivity Scale. Locker's single-item global self-rating of oral health was used to obtain information on self-rated oral health. Pearson correlation and negative binomial regression were used for data analysis. A higher negative affectivity score was associated with worse oral heath related quality of life, and a higher positive affectivity score was associated with better rating of oral health-related quality of life. On average, individuals who described their oral health as worse scored higher on Oral Health Impact Profile-14. Negative affectivity and positive affectivity influence individuals' perceptions of their oral health and quality of life. If possible, investigations of oral health-related quality of life measures should also include a brief personality assessment.

## 1. Introduction

Oral health-related quality of life (OHRQOL) is increasingly used to evaluate effectiveness and patient satisfaction with oral disease treatment [1, 2]. Many oral diseases such as periodontitis are related to a wide range of systemic diseases [3, 4]. Therefore, they affect the quality of life in many ways [5]. Although OHRQOL measures are considered to be strongly associated with clinical oral disease status and treatment outcomes, they only represent individuals' interpretation of health status. This interpretation is influenced by sociodemographic and personality characteristics [6], meaning that, after treatment, the same clinical signs may elicit different reactions in people with different personality characteristics [7]. Personality is also known to affect pain perception [8, 9].

Recently, personality measurement methods have been systematized and a consensus on the structure of adult personality traits has emerged [10]. This has merged into the oral health field and it could be important for oral health promotion activities [11].

The Positive Affectivity and Negative Affectivity Scale schedule (PANAS) is a brief, easy to use, reliable, and valid personality measurement method. It consists of two ten-item scales that describe different emotions related to positive affect (PA) and negative affect (NA) (two dominant dimensions of personality) [12]. Positive affect is the extent to which one feels enthusiastic, active, and alert, whereas negative affect includes a variety of negative mood states such as anger, contempt, and disgust. PA and NA are similar to the personality traits of extraversion and neuroticism, respectively [12]. Thomson et al. suggested that the PANAS could be useful if concurrently used with a self-report oral health measure in studies related to quality of oral health [11].

A study carried out on Japanese older people observed an association between personality traits such as extraversion and neuroticism and OHRQOL independently of present clinical measures [1]. Another study concluded that personality traits such as neuroticism influence self-rated quality of life in patients with oral mucosal disease [2]. Also, Kressin and colleagues reported poorer OHRQOL in depressed adults of USA [13]. Moreover another study in Iran revealed an association between type *D* personality (tendency towards negative affectivity) and OHRQOL in cleft lip and palate patients [14].

Recently published studies regarding this issue have stated that better self-reported oral health was associated to higher life satisfaction [15] or some types of personality traits such as consciousness [16]. Thompson et al. also found that negative personality aspects were associated to worse oral health-related quality of life [17].

The widespread use of self-rated oral health measures in oral health services research means that making any judgment of the efficacy of treatments or planning any service according to health states driven from such measures should consider interpersonal differences. Likewise, knowing the relationship between personality characteristics and self-reported oral health could be useful when interpreting data on patients' satisfaction levels. Accordingly, this study aimed to determine whether an association existed between self-reported OHRQOL and negative and positive personality traits.

## 2. Methods

This cross-sectional assessment was carried out on 443 adult participants, 17–50 years of age. This study received ethical approval from Kerman University of Medical Science ethical committee (KA 930328). Participants were recruited from Kerman University of Medical Sciences dental clinic. This is a public dental clinic that provides all types of oral and dental health care. In morning shifts, treatment is carried out by undergraduate and postgraduate dental students with lower cost. Therefore, usually patients with lower socioeconomic status refer to this clinic in morning shifts. In afternoon shifts, dental specialists carry out dental treatment with higher costs; thus, afternoon referents are usually from higher socioeconomic status. A convenience sampling method was used to recruit volunteer participants from morning and afternoon shifts. Written informed consent was obtained before data collection. Participants were asked to complete a questionnaire.

Demographic data such as gender, educational status, and their living place (rural, urban or suburbs) were recorded. Locker's global self-rating oral health measure was used to rate individuals' oral health. The question was “How would you describe the health of your teeth and mouth?”, used with the response categories of “poor”, “fair”, “good”, “very good,” or “excellent”. One option was selected among the five answering options.

Also a single YES/NO question determined whether participants had lost a tooth or not.

The short version of the oral health impact profile (OHIP-14) was used to assess participants' OHRQOL. A valid and reliable version of OHIP-14 is available for Persian speaking individuals [18]. Participants were asked how frequently they had experienced different impacts of oral health in the previous 12 months. Responses were recorded as follows: 0 = never, 1 = hardly ever, 2 = occasionally, 3 = fairly often, and 4 = very often.

The Positive Affectivity Negative Affectivity Scale (PANAS) was used to assess positive and negative personality traits. This scale comprises two 10-item scales representing positive emotionality and negative emotionality [12]. Participants were asked about how much they had experienced specific feelings in the past year for positive affect (interested, excited, strong, enthusiastic, proud, alert, inspired, determined, attentive, and active) and negative affect (distressed, upset, guilty, scared, hostile, irritable, ashamed, nervous, jittery, and afraid). Responses were recorded as follows: very slight or not at all, a little, moderate, quite a bit, and extreme. The internal consistency reliability (coefficient alpha) was also measured for PAS and NAS.

Pearson's *r* was used to determine the correlation between OHIP-14 and negative affectivity and positive affectivity scores. The OHIP score is a counting variable; therefore, negative binominal regression was used to determine factors associated with the OHIP score. Results were presented as crude incidence risk ratio (IRR) and adjusted IRR.

## 3. Results

Of the 443 people who participated in the study, 33.2% were men and 66.8% were women. Mean age of participants was 32.4 (sd 9.1). Basic demographic data of participants are listed in Table 1.

Internal reliability of OHIP questions was calculated as Cronbach's Alpha of 0.90. The mean overall OHIP score was 18.0 (sd 11.4). Mean OHIP score of male participants was 18.7 (sd 12.2), and mean OHIP score of female participants was 17.6 (sd 11.0).

For Positive Affectivity and Negative Affectivity Scale, internal consistency reliability (Cronbach's Alpha) was calculated as 0.688 and 0.684, respectively. Mean positive affect scale score of participants was 30.3 (sd 5.6), and mean negative affect scale score was 26.9 (sd 6.1).

The correlation matrix of OHIP-14 score and PANAS scores is presented in Table 2.

OHIP-14 score had a negative and statistically significant correlation with positive affect scale score (PAS) (Pearson *r* = −0.20, *p* < 0.01). Also, the correlation between OHIP-14 score and negative affect scale score (NAS) was positive and statistically significant (Pearson *r* = +0.10, *p* < 0.05). The positive affect scale score and negative affect scale score were also significantly and negatively correlated (Pearson *r* = −0.04, *p* < 0.01) (Table 2).

The OHIP score is a counting variable; therefore, negative binominal regression was used to determine factors associated with the OHIP score. Results are presented as crude incidence risk ratio (IRR) and adjusted IRR in Table 3.

In the crude model, the OHIP-14 score was significantly associated with age. Those who were older than 35 had significantly higher OHIP scores than those in the reference group (16–24 years).

“Losing a teeth” was also significantly associated with the OHIP score. The OHIP score of participants who had at least one lost tooth was 1.38 times higher than those with no lost teeth.

Regarding oral health self-assessment, participants who described their oral health better had lower OHIP-14 scores. For participants who described their oral health as good, IRR was 0.65 which means, OHIP score is 35% (1–0.65 = 0.35) lower than those who described their oral health as poor (reference group).

PAS was also significantly associated with OHIP-14 score (IRR_crude_ = 0.98) which means with a one unit increase of PAS, OHIP score decreases by 2% (1–0.98 = 0.02).

NAS was also significantly associated with the OHIP-14 score in the crude model.

After adjusting for all variables, self-assessment of oral health and PAS were significantly associated with the OHIP-14 score (Table 3).

The comparison of crude and adjusted results revealed that after adjusting for all variables; some variables lost their significant association with OHIP-14 score. The backward method was used to obtain a more accurate result. First, all variables were put in the model. Then, variables with the highest *p* value were eliminated from the model. This was continued until all variables were significant. Results are presented in Table 4.

Final results after the backward method revealed that losing a tooth, oral health self-assessment, PAS, and NAS were associated with the OHIP-14 score. Participants who had lost a tooth had an OHIP score that was 1.31 times higher than those who had not lost a tooth. Participants who described their oral health as good had an IRR of 0.7 which shows that their OHIP-14 score was (1–0.7 = 0.3) less than those who described their oral health as poor.

Also, results show that with a one unit increase in PAS, OHIP-14 score was (1–0.98 = 0.02) less. And, with a one unit increase of NAS, OHIP-14 score was 1.01 times higher.

## 4. Discussion

Findings of the study revealed that both positive and negative personality traits of people influenced patients' self-expressed oral health-related quality of life. In terms of personality characteristics, people who were on average, more interested, excited, strong, enthusiastic, proud, alert, inspired, determined, and attentive reported better OHRQOL. Conversely, people who had felt more distressed, upset, guilty, scared, hostile, irritable, ashamed, nervous jittery, and afraid had worse OHRQOL. Although personality was assessed using different methods, similar results have been obtained from research concerning oral mucosal disease patients, prosthetic patients, and implant and malocclusion patients. In all these researches, the NEO-FFI was used to assess 5 domains of personality and neuroticism was found to be related to patients' OHRQOL after different treatments [2, 19–22]. Neuroticism is a personality domain which presents a wide range of negative feelings [23] similar to NAS emotions examined in this study. Abu Hantash discovered that neuroticism, openness, consciousness, and agreeableness are helpful in predicting patient satisfaction with implant supported dentures after treatment [21].

An important factor influencing oral health-related quality of life is the pain experienced by patients before and even after dental treatments; therefore, pain tolerance is important in patients' quality of life. Many studies have reported negative personality traits to be associated with higher pain perception [8, 9] which could be the reason for lower quality of life.

Another study carried out on patients who had received prosthetic rehabilitation treatment presented neuroticism as the only personality dimension to be related to OHRQOL-UK, OHIP and DIDL (dental impact on daily living). Patients with higher neuroticism scores reported lower quality of life and higher impacts on oral health and lower total satisfaction with their denture [20].

Over all, there seems to be a high consensus on the negative influence of negative personality traits on oral health quality of life. Although some research as in this study, have reported positive affectivity to be related to oral heath quality of life [24], but results concerning positive personality traits are still controversial and more research is needed to determine the effect of positive emotions on oral health-related quality of life.

Results of this study showed that OHIP was related to the single-item oral health self-rating question. This means that a single question regarding self-rating oral health could predict OHRQOL. This finding was consistent with that of the previous studies [25–29]. A single self-rating oral health question is easier and less time-consuming to answer compared to multiple questions in various oral health-related quality of life questionnaires such as OHIP. Therefore, if such one question can predict OHRQOL (as in this study), it would be extremely useful in extended epidemiological studies to replace long and tiresome questionnaires.

In this study population, people who had lost at least one tooth, had a lower oral health-related quality of life compared to those who had not lost a tooth. Other studies have reported similar results [30, 31].

Our work clearly has some limitations. Although the sampling was performed in a dental clinic who has referents from a wide range of socioeconomic status, due to the convenient sampling method, the findings may not be totally generalizable to the population.

Despite all limitations, to our knowledge, this paper is the first to use PANAS in order to investigate the association between OHRQOL and personality traits.

This paper highlights the importance of considering different personality traits in studies related to OHRQOL. Also, it is important that clinicians be familiar with different aspects of personality traits. This could help them better understand and interpret self-reported oral health towards gaining better treatment results. Assessing personality traits before treatment may be useful for clinicians when interpreting patients' reported health outcomes which may lead to a better communication between the patient and clinician [8]. Sometimes, along with the actual dental treatment, psychological support would be necessary to maintain patient cooperation and help achieve the highest satisfaction level after treatment.

## 5. Conclusion

Over all, people who had experienced higher levels of positive emotions reported higher oral-health-related quality of life and people who experienced negative emotions expressed lower oral health-related quality of life. Therefore, personality characteristics should be considered when assessing treatment outcomes of oral disease.

## Figures and Tables

**Table 1:** Demographic characteristics of participants.

		Male		Female	
		Frequency	Valid percentage	Frequency	Valid percentage
Employment status	Not employed	54	37.5	185	64
	Employed	90	62.5	104	36
	Total	144	98.6	289	100

Education	Primary school	34	25	26	10
	High school	41	30.1	110	42.5
	College	20	14.7	46	17.8
	Higher	41	30.1	77	29.7
	Total	136	100	259	100

Living area	Urban	134	92.4	256	87.4
	Suburban	6	4.1	22	7.5
	Rural	5	3.4	15	5.1
	Total	145	100	293	100

**Table 2:** Correlation matrix of OHIP-14 scores and PAS score and NAS score (Pearson's correlation (*r*)).

	OHIP-14 score	Positive affect scale score (PAS)
Positive affect scale score (PAS)	−0.20^*∗∗*^*p* value = 0.001	1
Negative affect scale score (NAS)	+0.10^*∗*^*p* value = 0.024	−0.04^*∗∗*^*p* value

^*∗∗*^Correlation is significant at the 0.01 level. ^*∗*^Correlation is significant at the 0.05 level.

**Table 3:** The association between OHIP 14 score, PAS, NAS, and demographic characteristics.

Variables		Crude IRR	*p* value	Adjusted IRR	*p* value

Age	16–24	Reference			
	25–34	1.00 (0.83, 1.20)	0.974	0.95 (0.078, 1.17)	0.662
	35–44	1.14 (0.92, 1.40)	0.224	1.05 (0.82, 1.34)	0.686
	>45	1.40 (1.09, 1.79)	0.008^*∗*^	1.25 (0.94, 1.67)	0.129

Sex	Male	Reference			
	Female	0.99 (0.85, 1.15)	0.882	0.97 (0.82, 1.16)	0.768

Employment status	Not employed	Reference			
	Employed	0.96 (0.83, 1.11)	0.558	0.98 (0.83, 1.16)	0.858

Education	Primary school	Reference			
	High school	0.92 (0.73, 1.16)	0.477	0.90 (0.70, 1.14)	0.384
	College	0.82 (0.82, 1.41)	0.591	1.14 (0.86, 1.50)	0.359
	Higher	0.84 (0.66, 1.07)	0.155	0.91 (0.71, 1.18)	0.490

Living area	Urban	Reference			
	Suburban	1.24 (0.92, 1.66)	0.152	1.28 (0.93, 1.76)	0.130
	Rural	1.14 (0.81, 1.61)	0.454	1.15 (0.79, 1.66)	0.462

Losing any tooth	No	Reference			
	Yes	1.38 (1.15, 1.66)	<0.0001^*∗∗*^	1.18 (0.96, 1.45)	0.120

Self-assessment of oral health	Poor	Reference			
	Fair	0.75 (0.63, 0.90)	0.002^*∗∗*^	0.81 (0.66, 0.99)	0.041^*∗*^
	Good	0.65 (0.54, 0.77)	<0.0001^*∗∗*^	0.70 (0.52, 1.08)	<0.0001^*∗∗*^
	Very good	0.61 (0.44, 0.86)	0.004^*∗∗*^	0.75 (0.52, 1.08)	0.123
	Excellent	0.82 (0.50, 1.34)	0.426	0.94 (0.55, 1.58)	0.806
PAS	—	0.98 (0.97, 1.00)	0.004^*∗∗*^	0.98 (0.97, 1.00)	0.033^*∗*^
NAS	—	1.01 (1.00, 1.02)	0.041^*∗*^	1.01 (0.99, 1.02)	0.131

^*∗∗*^Correlation is significant at the 0.01 level. ^*∗*^Correlation is significant at the 0.05 level.

**Table 4:** Final modelling of OHIP-14 score based on associated variables using the backward method.

Variables	IRR (95% CI)	*p* value
Losing a tooth		
No	Reference	
Yes	1.31 (1.10, 1.57)	0.003^*∗∗*^

Self-assessment of oral health		
Poor	Reference	
Fair	0.81 (0.67, 0.97)	0.021^*∗*^
Good	0.70 (0.59, 0.84)	<0.0001^*∗∗*^
Very good	0.76 (0.54, 1.07)	0.122
Excellent	0.88 (0.54, 1.43)	0.601
PAS	0.98 (0.97, 0.99)	0.028^*∗*^
NAS	1.01 (1.00, 1.02)	0.035^*∗*^

^*∗∗*^Correlation is significant at the 0.01 level. ^*∗*^Correlation is significant at the 0.05 level.

## Data Availability

The datasets used and/or analyzed during the current study are available from the corresponding author on reasonable request.
